# Secreted aspartyl protease 3 regulated by the Ras/cAMP/PKA pathway promotes the virulence of *Candida auris*

**DOI:** 10.3389/fcimb.2023.1257897

**Published:** 2023-09-15

**Authors:** Ji-Seok Kim, Kyung-Tae Lee, Yong-Sun Bahn

**Affiliations:** ^1^ Department of Biotechnology, College of Life Science and Biotechnology, Yonsei University, Seoul, Republic of Korea; ^2^ Korea Zoonosis Research Institute, Jeonbuk National University, Iksan, Jeonbuk, Republic of Korea

**Keywords:** *C. auris*, Ras/cAMP/PKA signaling pathway, secreted aspartyl proteinase, Sapa3, virulence, a human fungal pathogen

## Abstract

The surge of multidrug-resistant fungal pathogens, especially *Candida auris*, poses significant threats to global public health. *Candida auris* exhibits resistance to multiple antifungal drugs, leading to major outbreaks and a high mortality rate. With an urgent call for innovative therapeutic strategies, this study focused on the regulation and pathobiological significance of secreted aspartyl proteinases (SAPs) in *C. auris*, as these enzymes play pivotal roles in the virulence of some fungal species. We delved into the Ras/cAMP/PKA signaling pathway’s influence on SAP activity in *C. auris*. Our findings underscored that the Ras/cAMP/PKA pathway significantly modulates SAP activity, with PKA catalytic subunits, Tpk1 and Tpk2, playing a key role. We identified a divergence in the SAPs of *C. auris* compared to *Candida albicans*, emphasizing the variation between *Candida* species. Among seven identified secreted aspartyl proteases in *C. auris* (Sapa1 to Sapa7), Sapa3 emerged as the primary SAP in the pathogen. Deletion of Sapa3 led to a significant decline in SAP activity. Furthermore, we have established the involvement of Sapa3 in the biofilm formation of *C. auris*. Notably, Sapa3 was primarily regulated by Tpk1 and Tpk2. Deletion of *SAPA3* significantly reduced *C. auris* virulence, underscoring its pivotal role in *C. auris* pathogenicity. The outcomes of this study provide valuable insights into potential therapeutic targets, laying the groundwork for future interventions against *C. auris* infection.

## Introduction

The emergence of multidrug-resistant pathogenic fungi has precipitated a severe global public health crisis, with more than 150 million individuals affected by serious fungal infections annually, leading to approximately 1.7 million fatalities (Kainz et al., 2020). Since 2009, *Candida auris*, a multidrug-resistant pathogenic fungus, has caused outbreaks of candidemia in healthcare settings worldwide (Satoh et al., 2009). This organism exhibits significant resistance to existing antifungal drugs, especially azoles; for instance, fluconazole resistance rates exceed 90% among strains found in the United States (Ahmad and Asadzadeh, 2023). This underscores an urgent need for the development of innovative therapeutic strategies to combat this formidable fungal pathogen.

*Candida auris*, a transmissible pathogenic fungus, poses a significant threat due to its potential to cause invasive and bloodstream infections (Mohsin et al., 2020). The associated mortality rates range from 30% to 60%, underlining its lethal nature. The clinical presentation of invasive *C. auris* infections closely mirrors that of other *Candida* species, which complicates an early and accurate diagnosis (Ahmad and Alfouzan, 2021). Recognizing this, the Centers for Disease Control and Prevention (CDC) have elevated their concern regarding *C. auris*. Echinocandins are currently the primary treatment option for patients with *C. auris* infections, often paired with amphotericin B or isavuconazole in combination therapy (Ruiz-Gaitan et al., 2019). Alarmingly, there has been a marked increase in concurrent *C. auris* infections among immunocompromised individuals, especially those afflicted by COVID-19 (Khojasteh et al., 2022). This trend underscores the urgent need for a more comprehensive understanding of *C. auris* infection mechanisms and pathogenicity.

Hydrolytic enzymes, including secreted aspartyl proteinases (SAPs), play a significant role in the pathogenicity of the opportunistic pathogen *Candida albicans* (Schaller et al., 2005). The proteinases of *C. albicans* encompass a variety of isoenzymes, encoded by at least 10 distinct SAP genes. Differential regulation of these genes has been demonstrated *in vitro*, suggesting that individual SAP genes may serve specific functions during various stages of infection and in different types of infections (Naglik et al., 2003). Among the SAPs, Sap2 stands outs as a key SAP and serves as an enzyme utilizing proteins as a nitrogen source (Hube et al., 1997). Deletion of Sap2 in *C. albicans* leads to a significant reduction in SAP activity, with previous studies noting the nearly attenuated phenotype of *sap2*Δ mutants (Hube et al., 1997). In addition, *sap4*Δ *sap5*Δ *sap6*Δ mutants display attenuated virulence (Sanglard et al., 1997). Like *C. albicans*, *C. auris* also exhibits SAP activity (Wang et al., 2018). Despite some variation between strains, the majority have been found to demonstrate SAP activity (Bing et al., 2022). This activity is significantly more pronounced at 37°C compared to 25°C (Bing et al., 2022). An additional trend observed is that *MTL*a isolates generally exhibit higher SAP activity levels than *MTL*α isolates (Bing et al., 2022).

The expression of SAPs in *C. albicans* is controlled by multiple signaling pathways and transcription factors. Among these, the MAPK signaling pathway, particularly via Cph1, positively influences the expression of *SAP4*, *SAP5*, and *SAP6*, all of which contribute to hyphal development (Naglik et al., 2004). Conversely, the expression of *SAP6* and *SAP7* is negatively regulated by Tup1, whereas *SAP9* is negatively controlled by both Tup1 and Mig (Naglik et al., 2004). Furthermore, the cAMP signaling pathway plays a role in SAP regulation (Naglik et al., 2004). It is worth noting that Efg1, activated by the PKA catalytic subunit, positively regulates *SAP1*, *SAP3*, *SAP4*, *SAP5*, and *SAP6* (Naglik et al., 2004).

Our recent work shed light on the pathobiological functions of the Ras/cAMP/PKA pathway in *C. auris* (Kim et al., 2021; Kim et al., 2023). We found that hyperactivation of the Ras/cAMP/PKA signaling pathway results in diminished virulence in *C. auris*. In particular, we observed a decline in heat resistance following the deletion of Bcy1 and Pde2 (Kim et al., 2023). Intriguingly, this correlates with a decrease in intracellular glycogen accumulation, reducing survival rates in nutrient-starved environments (Kim et al., 2023). The inability to persist within the host over extended periods contributes to a decrease in virulence. On the other hand, we discovered that the deletion of Cyr1, Tpk1, Tpk2, and Ras1, which inactivates the Ras/cAMP/PKA signaling pathway, does not lead to a decrease in virulence (Kim et al., 2021; Kim et al., 2023). However, the connection between the Ras/cAMP/PKA signaling pathway and SAP activity remains largely uncharted in *C. auris*, and the specific aspartic proteinase enzyme involved in this process is still unknown.

In this study, we performed a detailed analysis of SAP activity in *C. auris*, investigating its regulation by the Ras/cAMP/PKA signaling pathway. We aimed to identify the major SAP genes involved and elucidate the role of SAP activity in the pathogenicity of *C. auris*. Our results confirmed that SAP activity in *C. auris* is regulated by the Ras/cAMP/PKA signaling pathway, primarily controlled by the PKA catalytic subunits. Interestingly, we found that Sapa3 is the major SAP in *C. auris*. We further substantiated the role of SAP activity as a significant virulence factor contributing to *C. auris* pathogenicity. Our work provides a comprehensive understanding of the regulatory mechanisms and pathobiological significance of SAP activity in *C. auris*. The insights gained hold valuable implications for the development of novel therapies targeting this pathway, potentially enhancing the treatment of candidiasis.

## Materials and methods

### Ethics statement

Animal care and research were approved after deliberation by the Institutional Animal Care and Use Committee of the Experimental Animal Center at Jeonbuk National University. (Approval number: JBNU 2022-092). All experiments followed the experimental ethics guidelines. Animal experiments were conducted at the Core Facility Center for Zoonosis Research (Jeonbuk National University, South Korea).

### *Candida auris* strains and growth media

*Candida auris* strains used in this study are listed in **Table S1** in the **Supplemental Material**. The parental wild-type strain, B8441 (AR0387), was obtained from the Centers for Disease Control and Prevention (CDC). These isolates and the constructed mutant strains were stored as frozen stocks in 20% glycerol at -80°C until further use. Yeast strains were routinely cultured on YPD agar plates (2% agar in YPD broth: 1% yeast extract, 2% peptone, and 2% D-glucose) at 30°C for 24-48 hours. For liquid cultures, cells were grown in YPD broth at 30°C with shaking at 200 rpm. For experimental assays, cells were inoculated into fresh YPD broth and grown to mid-log phase (an optical density at 600 nm (OD_600_) of 0.8) before being subjected to various treatments.

### Gene deletion and complementation

To generate gene deletion mutants, we used the nourseothricin resistance marker (*CaNAT*) flanked by 0.3- to 0.7-kb 5′ and 3′ regions of each target gene, including *SAPA1*, *SAPA2*, *SAPA3*, *SAPA4*, *SAPA5*, *SAPA6*, and *SAPA7*. Each gene disruption cassette containing a selection marker was constructed using double-joint PCR. To amplify the flanking regions of a target gene, we used L1-L2, and R1-R2 primer pairs in the first round of PCR. The *CaNAT* selection marker was amplified by PCR using the plasmid pV1025 containing the *CaNAT* gene as a template and the primer pairs listed in **Table S1** in the **Supplemental Material**. The first round of PCR products of the flanking regions and *CaNAT* marker were purified together and used as templates for the second round of double-joint PCR. In the second round of PCR, 5′- and 3′- gene disruption cassettes containing split *CaNAT* selection markers were amplified by L1-split primer 2 and R2-split primer 1, respectively (**Table S1** in the **Supplemental Material**).

For the transformation of *C. auris with* gene disruption cassettes, we used a lithium acetate/heat-shock protocol with modifications. Cells were cultured overnight at 30°C in 50 mL YPD broth with shaking. We centrifuged 1.2 mL of cultured cells, washed them with dH_2_O and lithium acetate buffer (100 mM lithium acetate, 10 mM Tris, 1 mM EDTA, pH 7.5), and resuspended them in 300 μL of lithium acetate buffer. The transformation was set up with 10 μL of denatured salmon sperm DNA (Sigma, cat no. D9156), 100 μL of the competent cells, 500 μL of 50% PEG4000 (Sigma, cat no. P4338), and 50 μL of the amplified gene deletion cassette. The transformation mixture was incubated at 30°C for 6 hours with intermittent vortexing. Subsequently, the cells were subjected to a 20-minute heat shock at 42°C followed by 1 minute of cooling on ice. The cells were then pelleted, resuspended in 1 mL of YPD medium, and incubated at 30°C for 1 hour with shaking. After the incubation, the cells were washed twice with fresh liquid YPD medium and then spread onto selective YPD agar plates supplemented with 600 µg/mL nourseothricin. The plates were then incubated at 37°C for 2 days. We confirmed the desired genotype of each positive nourseothricin-resistant transformant by diagnostic PCR and Southern blot (**Figure S1**).

To confirm the phenotypes of the *sapa3*Δ mutant, we constructed corresponding complemented strains, in which each wild-type allele was re-integrated into its native locus. To generate each full-length gene fragment, Phusion-PCR was performed using genomic DNA from the wild-type B8441 strain as a template and each primer pair listed in **Table S1** in the **Supplemental Material**. The resulting fragments were directly cloned into the TOPO vector (Invitrogen) to generate the plasmids pTOP-SAPA3. After confirming the target sequence, the *CaHYG* inserts were sub-cloned into each pTOP vector to produce the pTOP-SAPA3-HYG. For the targeted re-integration into its native locus, pTOP-SAPA3-HYG was linearized by StuI, and introduced into each mutant by the lithium acetate heat-shock method. The correct genotype of the complemented strain was confirmed by diagnostic PCR (**Figure S1**).

### Secreted aspartyl proteinase activity assay

SAP activity was tested using the YCB-BSA method with slight modifications. *C. auris* strains were cultured overnight in 2 mL YPD broth at 30°C, washed, and resuspended in 1 mL of dH_2_O. Next, 3 μL of the suspended cells were then spotted on YCB-BSA plates (containing 23.4g yeast carbon base per liter and 0.2% BSA) and incubated at different temperatures for 3 days. The thickness of the halo was measured to determine the SAP activity, and the experiment was biologically repeated three times.

### Crystal violet assay

Cells of *C. auris* wild-type and mutant strains were cultured overnight at 30°C in 2 mL YPD liquid medium, washed twice with H_2_O, and then resuspended in MOPS-buffered RPMI-1640 media (pH 7.4 with 0.165 M MOPS and 2% glucose). For the crystal violet assay, the cell suspension was prepared with a concentration equivalent to an OD_600_ of 0.5. Subsequently, 200 μL of the cell suspension was loaded into each well of a 96-well plate, each inhibitor was treated at a concentration of 10 μM, and the cultures were incubated at 37°C and 220 rpm for 1 day. The next day, the cell suspension was completely removed, and the samples were completely dried in a dry oven at 65°C. To each well, 150 μL of a 0.3% crystal violet solution was added, and the samples were stained at room temperature for 10 minutes. Subsequently, the crystal violet solution was completely removed, and the wells were washed three times with ddH_2_O. The samples were then dried in a dry oven at 65°C. Next, 200 μL of 33% acetic acid was added to each well, and the crystal violet was dissolved completely for 1 minute. The solution was transferred to a clean 96-well plate, and the absorbance was read at OD_595_.

### Pseudohyphae induction

Cells were cultured overnight at 30°C in YPD medium and subcultured to OD_600 _of 0.8 in fresh YPD medium. After sub-culturing the cells were suspended in YPD broth supplemented with 100 mM HU and incubated at 30°C for 24 hours. After hyphal growth, each sample was fixed using a 10% formalin solution and stained with a calcofluor white solution. The samples that had been fixed and stained were then incubated in a dark environment for 30 minutes before being photographed.

### Growth and stress sensitivity spot assay

To analyze the growth and sensitivity to various stresses of WT and mutant strains. To do this, *C. auris* cells grown overnight at 30°C were serially diluted tenfold, four times (final dilution 1:104) and spotted onto YPD plates. For growth measurements, plates were incubated at 30°C, 37°C, and 42°C. Growth was assessed qualitatively by photographing the plates 1 day later.

Various stresses were imposed by adding to the media chemical agents that would impose stress on the cells. Osmotic stress was supplied as sorbitol. Cation and salt stress were imposed with NaCl or KCl. Oxidative stress was supplied with hydrogen peroxide, tert-butyl hydroperoxide (an organic peroxide), menadione (a superoxide anion generator), or diamide (a thiol-specific oxidant). Membrane destabilizing stress was imposed with SDS and cell-wall destabilizing stress was imposed with CR, fluconazole, amphotericin B, or caspofungin for antifungal drug susceptibility. Cells were incubated at 30°C and photographed 1 to 4 days after treatment.

### Virulence study

To evaluate the virulence of *C. auris* wild-type and Ras/cAMP/PKA mutant strains *in vivo*, we used a murine systemic infection model with reference to previous studies (Gao et al., 2021; Kim et al., 2021). Fungal cells were incubated overnight at 30°C in YPD broth and washed three times with PBS. Cell concentrations were measured and adjusted to 10^8^ cells/mL in PBS. To confirm colony-forming units (CFUs) and viability of the inoculum, the diluted cells were plated onto YPD agar plates and incubated at 37°C for 24 h. SPF/VAF-confirmed inbred 6-week-old female mice of the BALB/c (AnNCrlOri) strain were used for this study (ORIENT BIO INC., South Korea), and they were habituated for one week before the experiment. For infection, the mice were restrained, and their tails were placed in warm (40°C) water to expand the lateral veins. The 100 µL of cell suspension was injected intravenously. Daily monitoring of survival was performed and abnormal behavior (head tilt or body spinning) was judged as a symptom of infection (Gao et al., 2021), and the mice were sacrificed as a humane endpoint for the experiment. The survival curves were analyzed using the Log-rank (Mantel-Cox).

### Total RNA preparation and quantitative RT-PCR

Total RNA was extracted from *C. auris* wild-type and Ras/cAMP/PKA mutant strains cultured overnight at 30°C in YPD broth. Briefly, cells were collected by centrifugation after reaching an OD_600_ of 0.8, frozen in liquid nitrogen, and lyophilized. For stress conditions, 10 mL of the culture was sampled for the basal state, and the remaining 30 mL was further incubated with stress agents. Total RNA was isolated by the Trizol extraction method with Easy-blue (Intron). Complementary DNA (cDNA) was synthesized from purified total RNA using reverse transcriptase (Thermo Scientific). Quantitative PCR was performed using specific primer pairs for each gene and the CFX96TM Real-Time system (Bio-Rad). *ACT1* expression was used for normalization. Statistical analysis was performed using one-way ANOVA, followed by Bonferroni’s multiple-comparison test. All experiments were conducted in triplicate and repeated thrice biologically.

## Results

### The Ras/cAMP/PKA pathway regulates SAP activity in *C. auris*

**Figure 1:**
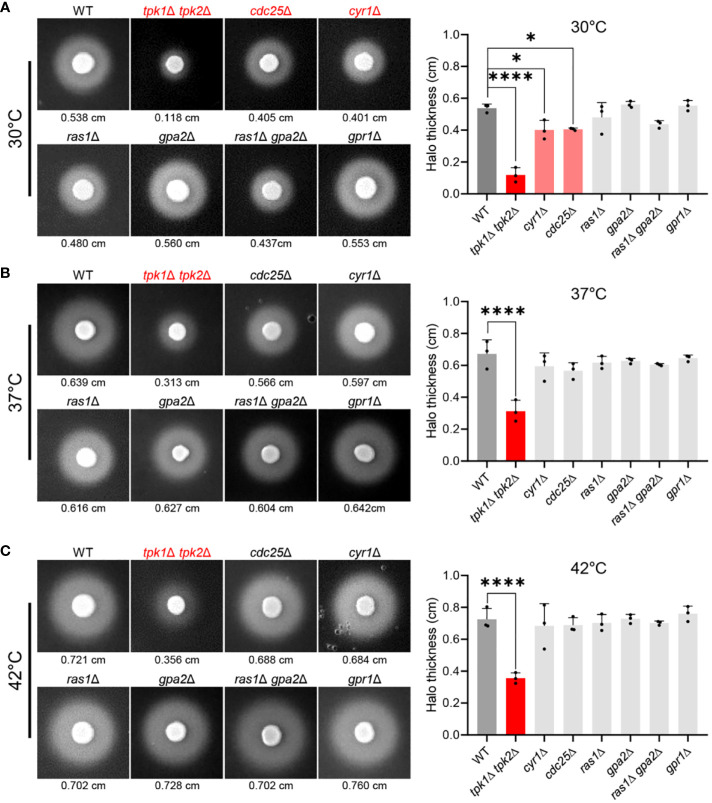
Control of SAP activity in *C*. *auris* by the Ras/cAMP/PKA signaling pathway. **(A–C)** A comparison of SAP activities of *C*. *auris* wild-type and Ras/cAMP/PKA mutant strains at various temperatures. Cells of the wild-type and mutants *tpk1*Δ *tpk2*Δ (YSBA24), *cdc25*Δ (YSBA61), *cyr1*Δ (YSBA21), *ras1*Δ (YSBA41), *gpa2*Δ (YSBA39), *ras1*Δ *gpa2*Δ (YSBA68), and *gpr1*Δ (YSBA46) were cultured overnight, washed twice with deionized water (dH_2_O), and resuspended in 1 mL dH_2_O. We spotted 3 µL of the resuspended cell onto solid YCB-BSA media and incubated them for 3 days at **(A)** 30°C, **(B)** 37°C, and **(C)** 42°C. The values indicated below each figure represent the average halo thickness. Three biologically independent experiments were performed and one representative data are shown here. Error bars indicate standard deviation. Statistical analysis was performed using one-way ANOVA with Bonferroni’s multiple-comparison test (*P < 0.05; ****P < 0.0001).

To elucidate the role of the Ras/cAMP/PKA pathway in modulating SAP activity in *C. auris*, we measured SAP activity in mutant strains where the Ras/cAMP/PKA pathway was inactivated. We assessed SAP activity in wild-type and mutant strains at 30°C, 37°C, and 42°C (**Figures 1A–C**). Compared to the wild-type strain, *tpk1*Δ *tpk2*Δ, *cdc25*Δ, and *cyr1*Δ strains exhibited a noticeable reduction in SAP activity at 30°C (**Figure 1A**). This reduction was evident in the measurements of average halo thickness, with *tpk1*Δ *tpk2*Δ, *cdc25*Δ, and *cyr1*Δ showing thicknesses of 0.118 cm, 0.405 cm, 0.401 cm respectively, compared to the wild-type’s 0.538 cm (**Figure 1A**). The *tpk1*Δ *tpk2*Δ strain, in particular, displayed a severe decrease in SAP activity across all temperature conditions. As the temperature increased, SAP activity in the *cdc25*Δ and *cyr1*Δ strains reached levels closer to the wild-type (**Figures 1B, C**). However, the *tpk1*Δ *tpk2*Δ strain consistently showed significantly lower SAP activity than the wild-type strain, even at higher temperatures. At 37°C, the average halo thickness of the wild-type strain was 0.639 cm, while *tpk1*Δ *tpk2*Δ exhibited a thickness of 0.313 cm (**Figure 1B**). At 42°C, the wild-type’s average halo thickness was 0.721 cm, whereas *tpk1*Δ *tpk2*Δ showed a thickness of 0.356 cm (**Figure 1C**).

Our findings indicate that like in *C. albicans*, the SAP activity in *C. auris* is temperature-dependent, providing additional evidence that the Ras/cAMP/PKA pathway regulates this activity. Notably, our data support the pivotal role of PKA catalytic subunits in modulating SAP activity in *C. auris*. However, the decreased dependence of the Ras/cAMP/PKA signaling pathway in regulating SAP activity with increasing temperature suggests the involvement of additional signaling pathways in SAP regulation. In *C. albicans*, the MAPK pathway, in tandem with the Ras/cAMP/PKA signaling pathway, modulates SAP gene expression (Naglik et al., 2004).

### Identification and phylogenetic analysis of SAPs in *C. auris*

**Figure 2:**
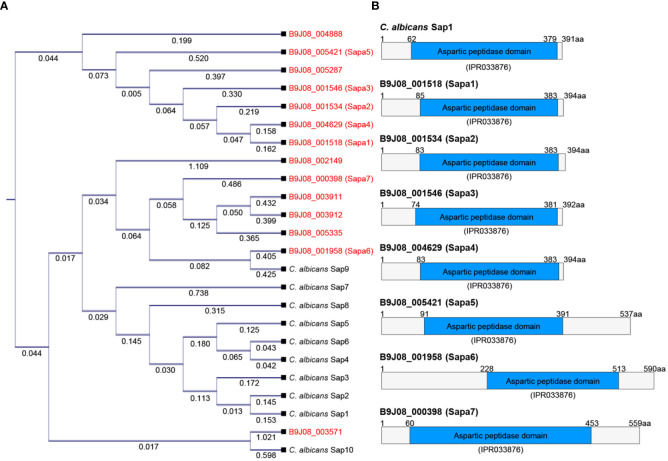
Comparison of SAP genes between *C*. *auris* and *C*. *albicans*. **(A)** Phylogenetic analysis of SAP genes. This phylogenetic tree was constructed in the form of a cladogram by aligning the amino acid sequences of SAP genes from *C*. *albicans* and *C*. *auris*. CLC Sequence Viewer 8.0 software was used for the analysis. **(B)** Protein domain analysis of SAP genes. This domain analysis image was created based on the domain information available on InterPro (https://www.ebi.ac.uk/interpro/).

To identify SAP-encoding genes in *C. auris*, we leveraged the *Candida* Genome Database, specifically looking for proteins containing the aspartic peptidase domain (IPR033876). Our search revealed that while *C. albicans* has 10 proteins with this domain, *C. auris* features 14 proteins. Phylogenetic analysis showed a notable divergence between the SAPs of *C. auris* and *C. albicans* (**Figure 2A**). A BLAST analysis performed on *C. albicans* SAPs against the *C. auris* genome revealed that Sap1, Sap2, Sap3, Sap4, Sap5, and Sap6 primarily aligned with B9J08_001518. Similarly, Sap7, Sap9, and Sap10 were most closely associated with B9J08_001958, and Sap8 first aligned with B9J08_001534 (**Table S2**). When we performed the reverse BLAST analysis using *C. auris* SAPs against the *C. albicans* genome, we found that B9J08_000398, B9J08_001958, B9J08_003911, B9J08_003912, and B9J08_005335 were best aligned with Sap9. Similarly, B9J08_001546, B9J08_004629, and B9J08_004888 primarily matched with Sap8. Furthermore, each of B9J08_005287, B9J08_001534, B9J08_005421, B9J08_001518, B9J08_002149, and B9J08_005371 demonstrated a first match with Sap5, Sap4, Sap3, Sap2, Yps7, and Arp1, respectively (**Table S2**).

Due to the overall low similarity between the SAPs of *C. auris* and *C. albicans* and the lack of one-to-one protein matches in the BLAST analysis, we decided to focus our investigations on the seven proteins with high scores and low E-values in the BLAST analysis of *C. albicans* SAPs against the *C. auris* genome. These proteins, labeled as Sapa1 to Sapa7 in *C. auris* (B9J08_001518: Sapa1, B9J08_001534: Sapa2, B9J08_001546: Sapa3, B9J08_004629: Sapa4, B9J08_005421: Sapa5, B9J08_001958: Sapa6, B9J08_000398: Sapa7), were selected for further analysis (**Figures 2A, B**). This outcome underscores the significant inter-species divergence within the aspartic peptidase domain.

### Sapa3 serves as a pivotal SAP in *C. auris*

**Figure 3:**
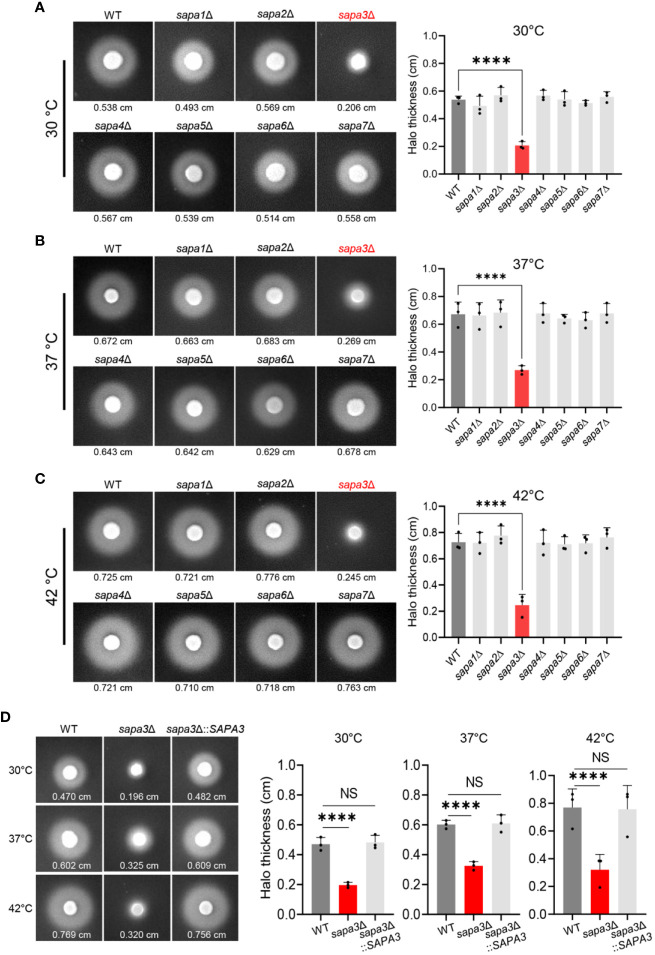
Role of Sapa3 as a key secreted aspartyl proteinase in *C*. *auris*. **(A–C)** Comparative analysis of SAP activities in wild-type and mutant strains of *C*. *auris* at different temperatures. Overnight cultured cells of the wild-type strain and SAP gene mutants, including *sapa1*Δ (YSBA77), *sapa2*Δ (YSBA81), *sapa3*Δ (YSBA119), *sapa4*Δ (YSBA135), *sapa5*Δ (YSBA125), *sapa6*Δ (YSBA91), and *sapa7*Δ (YSBA127), were washed with dH_2_O, resuspended in 1 mL of dH_2_O, and spotted onto solid YCB-BSA media using 3 µL of the cell suspension. The plates were incubated for 3 days at **(A)** 30°C, **(B)** 37°C, and **(C)** 42°C. The values below each figure represent the average halo thickness, indicative of SAP activity. Three biologically independent experiments were performed and one representative data are shown here. **(D)** SAP activities of *SAPA3* deletion mutant and complemented strains. Three biologically independent experiments were performed and one representative data are shown here. (A to D) Error bars indicate standard deviation. Statistical analysis was performed using one-way ANOVA with Bonferroni’s multiple-comparison test (****P < 0.0001; NS, not significant).

To elucidate the role of SAPs in *C. auris*, we generated knockout mutants for each of the seven SAP genes (**Figure S1**). Subsequently, we measured and compared the SAP activity of these mutants (*sapa1*Δ, *sapa2*Δ, *sapa3*Δ, *sapa4*Δ, *sapa5*Δ, *sapa6*Δ, and *sapa7*Δ) with the wild-type strain at 30°C, 37°C, and 42°C (**Figures 3A–C**). At 30°C, the wild-type strain demonstrated an average halo thickness of 0.538 cm. The *sapa1*Δ, *sapa2*Δ, *sapa4*Δ, *sapa5*Δ, *sapa6*Δ, and *sapa7*Δ mutants, with respective average halo thicknesses of 0.493 cm, 0.569 cm, 0.539 cm, 0.567 cm, 0.514 cm, and 0.558 cm, showed no significant deviation from the wild-type strain (**Figure 3A**). However, the *sapa3*Δ mutant manifested a pronounced decrease in SAP activity, with an average halo thickness of 0.206 cm, signifying a substantial reduction of about 61% (**Figure 3A**).

This pattern persisted across all temperature conditions. As the temperature rose, the SAP activity of the wild-type strain and all mutant strains, except *sapa3*Δ, escalated by roughly 30-40% compared to measurements at 30°C (**Figures 3B, C**). Yet, the *sapa3*Δ exhibited only a minimal increase in SAP activity. At 37°C, the wild-type strain displayed an average halo thickness of 0.672 cm, while *sapa3*Δ showed an average halo thickness of 0.269 cm − about 59% less than the wild-type (**Figure 3B**). Similarly, at 42°C, the wild-type registered an average halo thickness of 0.725 cm, while *sapa3*Δ showed a notable drop in SAP activity, with an average halo thickness of 0.245 cm, signifying a 66% decrease compared to the wild-type strain (**Figure 3C**).

To confirm the role of Sapa3, we generated the *sapa3*Δ::*SAPA3* complemented strain (**Figure S1**), and measured its SAP activity at 30°C, 37°C, and 42°C compared to the wild-type and *sapa3*Δ strains (**Figure 3D**). The complemented strain exhibited SAP activity levels equivalent to those of the wild-type strain (**Figure 3D**), affirming that *SAP3* encodes a major SAP in *C. auris* and that disruption of Sapa3 results in a loss of SAP activity irrespective of temperature. All these data emphasize the critical role of Sapa3 as the primary SAP in *C. auris*.

### Sapa3 is regulated by the Ras/cAMP/PKA pathway

**Figure 4:**
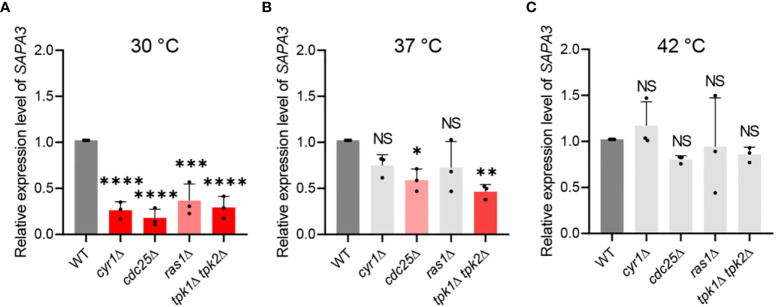
Regulation of *SAPA3* expression by the Ras/cAMP/PKA signaling pathway in *C*. *auris*. Quantitative reverse transcription PCR (qRT-PCR) analysis of *SAPA3* in wild-type and mutants *cyr1*Δ (YSBA21), *ras1*Δ (YSBA41), *cdc25*Δ (YSBA61), *and tpk1*Δ *tpk2*Δ (YSBA24). Cells were cultured overnight at 30°C in YPD broth, subcultured to OD_600 _ of 0.8 at **(A)** 30°C, **(B)** 37°C, and **(C)** 42°C in fresh broth, and extracted for total RNA. The expression level of *SAPA3* was normalized using *ACT1* as the standard. Three biologically independent experiments were performed. Error bars indicate standard deviation. Statistical analysis was performed using one-way ANOVA with Bonferroni’s multiple-comparison test (*P < 0.05; **P < 0.01; ***P < 0.001; ****P < 0.0001; NS, not significant).

Our findings highlight the regulatory role of the Ras/cAMP/PKA pathway on SAP activity and identify Sapa3 as the major SAP in *C. auris*. Subsequently, we investigated whether the Ras/cAMP/PKA pathway directly modulates Sapa3. At 30°C, inactivation of the Ras/cAMP/PKA pathway resulted in a significant decline in *SAPA3* expression (**Figure 4A**). Specifically, the *cyr1*Δ, *cdc25*Δ, *ras1*Δ, and *tpk1*Δ *tpk2*Δ strains displayed around 60-80% decrease in *SAPA3* expression compared to the wild-type strain (**Figure 4A**). As the temperature increased, however, the impact of the Ras/cAMP/PKA inactivation on *SAPA3* expression became less apparent. At 37°C, we observed a significant reduction in *SAPA3* expression in the *cdc25*Δ and *tpk1*Δ *tpk2*Δ strains, while the *cyr1*Δ and *ras1*Δ strains exhibited similar *SAPA3* expression levels to the wild-type strain (**Figure 4B**). At 42°C, the inactivation of the Ras/cAMP/PKA pathway did not lead to a decrease in *SAPA3* expression (**Figure 4C**). These results suggest that although *SAPA3* is a key SAP whose expression is modulated by the Ras/cAMP/PKA pathway, this regulation appears to diminish at higher temperatures. Notably, even at 42°C, the *tpk1*Δ *tpk2*Δ strain displayed decreased SAP activity. However, this was not coupled with a significant reduction in *SAPA3* expression compared to the wild-type strain, indicating the potential influence of other SAP genes on SAP activity in *C. auris*.

### Phenotypic analysis of SAP gene mutants

We next conducted phenotypic analyses to evaluate the role of SAP genes in biofilm formation, filamentous growth, and stress resistance in *C. auris*. Given that in *C. albicans*, SAP genes are implicated in biofilm development and pseudohyphae formation, we sought to ascertain if similar functionalities exist in *C. auris*.

**Figure 5:**
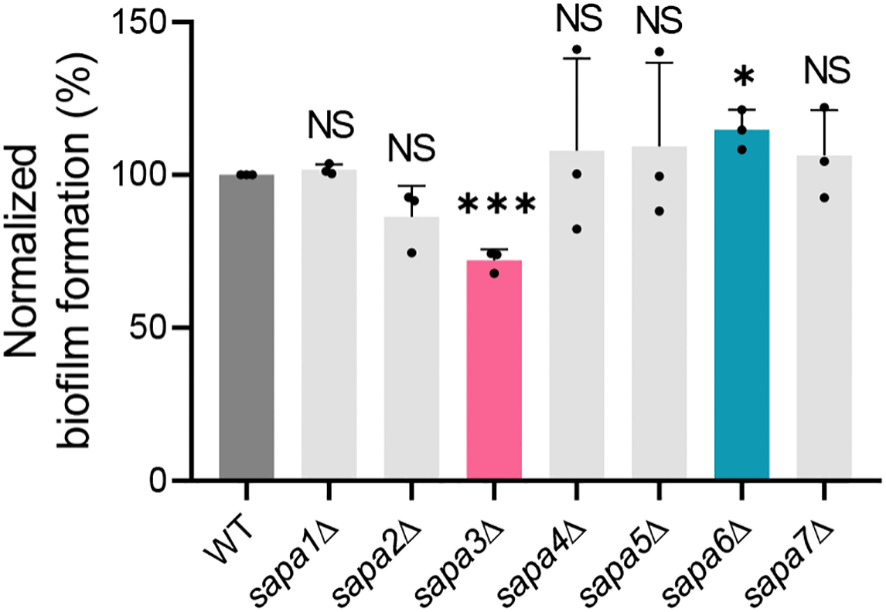
Role of Sapa3 in *C. auris* biofilm formation. Crystal violet staining was used to assess biofilm formation in *C. auris* wild-type and SAP mutant strains. The absorbance of the destaining solution for each strain was determined at 595 nm. Statistical significance was evaluated using the Student *t*-tests compared to the control (*P < 0.05; ***P < 0.001; NS, not significant).

Biofilm formation was gauged using the crystal violet assay. The biofilm-forming potential of the *sapa2*Δ mutant was marginally diminished by about 14% relative to the wild-type, though the difference did not reach statistical significance (**Figure 5**). On the other hand, the *sapa3*Δ mutant exhibited a 28% decrease in biofilm formation (**Figure 5**). Notably, the *sapa6*Δ mutant showed an increase in biofilm formation by around 14% compared to the wild-type (**Figure 5**). These observations underscore the significance of SAP genes in *C. auris* biofilm formation, especially spotlighting the integral function of Sapa3.

To probe into the pseudohyphae formation capability, the wild-type and mutant strains were incubated in 100 mM hydroxyurea over a 24-hour period. The outcome highlighted that all strains, including the wild-type, manifested standard pseudohyphae formation (**Figure S2**), suggesting that SAP genes are not critical for filamentous growth in *C. auris*. We also assessed the resistance of SAP gene mutants to a range of stress conditions. The wild-type and mutant strains exhibit similar growth patterns at 30°C, 37°C, and 42°C (**Figure S3**). Additionally, with the exception of a slightly diminished resistance of *sapa5*Δ to diamide and *sapa6*Δ to DTT, we observed no significant differences in resistance between wild-type and SAP gene mutants when exposed to various antifungal drugs, oxidative, osmotic, genotoxic, cell wall and membrane stresses, heavy metal challenges, ER stress, and acidic pH conditions (**Figure S3**). These results collectively indicate that SAP genes have a limited influence on the stress defense mechanisms in *C. auris*.

### Sapa3 is required for the pathogenicity of *C. auris*

**Figure 6:**
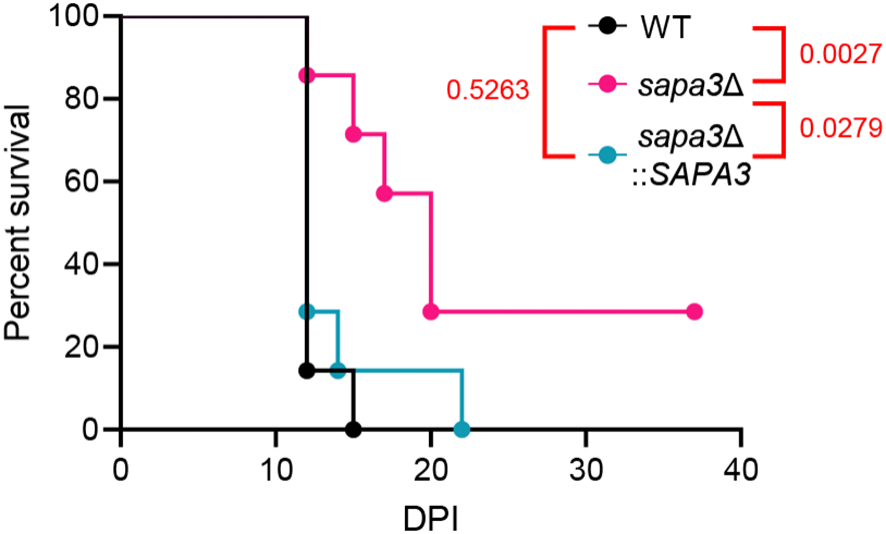
Sapa3 is involved in the pathogenicity of *C. auris*. 6-week-old female BALB/c mice were injected intravenously with 1 × 10^7^ cells of strains of *C. auris* wild-type and mutants. Survival was monitored daily, and statistical analysis was performed with Log-rank (Mantel-Cox) test by using Prism 8.0.

Next, we explored the contribution of Sapa3 to *C. auris* pathogenicity using and BALB/c mouse model. The mice were intravenously infected with the wild-type, *sapa3*Δ, and *sapa3*Δ::*SAPA3* strains, and their survival rate was monitored over 36 days. Compared to the wild-type and complemented strains, the *sapa3*Δ mutant manifested a significant decrease in pathogenicity (**Figure 6**). While all mice infected with the wild-type and complemented strains succumbed within 15 and 22 days, respectively (**Figure 6**), a portion of mice infected with the *sapa3*Δ mutant survived until day 36 (**Figure 6**). These results underscore the pivotal role of SAP as a key virulence factor in *C. auris*.

## Discussion

In this study, we focused on identifying the primary SAP gene, elucidating its regulatory mechanisms, and understanding its role in the pathogenicity of *C. auris*, a highly drug-resistant fungal pathogen. From our investigation, we made a significant discovery: the Ras/cAMP/PKA signaling pathway emerged as a primary regulator of SAP activity. Of the seven SAP genes (Sapa1 to Sapa7) investigated, Sapa3 was found to be the key player required for the virulence of *C. auris*. Importantly, the PKA catalytic subunits, Tpk1 and Tpk2, primarily regulated Sapa3. These findings have profound implications for understanding the pathogenesis of *C. auris* infections and suggest that targeting Sapa3 could be a promising strategy for treating *C. auris*-mediated candidiasis.

SAPs have been reported in various non-*Candida* fungal species, including *Aspergillus*, *Cryptococcus*, and *Mucor*, implying their potential roles in virulence (Gray et al., 1986; Lee and Kolattukudy, 1995; Pinti et al., 2007; Mandujano-Gonzalez et al., 2016). However, a thorough understanding of the functionality of these enzymes in these fungi remains elusive. In the context of *Candida* species, *C. albicans*, in particular, relies heavily on SAPs as primary virulence factors. Our homology analysis revealed that *C. albicans* SAP genes and *C. auris* SAP genes share a very limited similarity, except for a few genes. This suggests potential variations in the functions of SAPs among *Candida* species. Our study presents a comprehensive characterization of the genes associated with SAP activity in *C. auris*, emphasizing their vital role in virulence. Furthermore, we highlighted the regulatory role of the Ras/cAMP/PKA signaling pathway in modulating SAP activity, establishing its significance in both *C. albicans* and *C. auris*. These insights underline the need for further exploration of the regulation of SAP activity via the Ras/cAMP/PKA pathway in other pathogenic fungi.

Secreted proteinases are instrumental to the pathogenicity of *C. albican*s, with SAPs facilitating invasive infections by degrading a variety of human membranes (Naglik et al., 2003). Sap2, one of many proteinases in *C. albicans*, exhibits a broad range of activity. It targets protective elements such as mucin and secretory immunoglobulin A (sIgA) located on mucosal surfaces. Notably, Sap2 degrades sIgA, which typically prevents *C. albicans* from attaching to buccal epithelial cells (Naglik et al., 2003). Additionally, Sap2 in *C. albicans* is capable of degrading oral cavity constituents such as salivary proteins, keratin, and collagen, therefore undermining host defenses (Naglik et al., 2003). These findings underline the multifaceted activity of *C. albicans* Sap2 in modulating host-pathogen interaction and establish its crucial role in initiating infections (Naglik et al., 2003). As a result, the *sap2*Δ mutant strain manifests an almost avirulent phenotype, emphasizing the importance of Sap2 (Hube et al., 1997). Considering the mechanism of action of *C. albicans* Sap2, it is necessary to further investigate whether *C. auris* Sap3 contributes to pathogenicity by degrading various protective host surface components, such as mucin or sIgA, thereby weakening the host defense system. Alternatively, it would be equally significant if *C. auris* Sapa3 engages in pathogenicity through entirely different mechanisms.

The secretion pathway of SAPs in *C. albicans* has been extensively investigated, involving processes such as mRNA transfer, signal peptide cleavage, and Golgi-mediated processing (White and Agabian, 1995). The maturation of proenzymes is coordinated by the Kex2 proteinase, while the propeptide facilitates proper folding and zymogen inactivation (Newport and Agabian, 1997). Following encapsulation into secretory vesicles, SAPs are transported to the plasma membrane for integration into the cell wall or extracellular release. This sophisticated secretion pathway ensures the regulated delivery of SAPs, contributing to their physiological and pathogenic roles in fungi. Given the pivotal role of Kex2 in SAP maturation in *C. albicans*, it is crucial to explore whether a similar Kex2-mediated maturation of SAPs occurs in *C. auris*. If the involvement of Kex2 in *C. auris* is confirmed, strategies targeting Kex2 through deletion or inhibition could potentially attenuate the pathogenicity of *C. auris*.

Beyond *C. albicans*, several other *Candida* species, including *C. parapsilosis*, *C. tropicalis*, and *C. lusitaniae*, have been reported to possess proteolytic activity, specifically in degrading bovine serum albumin (BSA) (Dostal et al., 2003). However, *C. kefyr*, *C. krusei, C. glabrata*, and *C. guilliermondii* reportedly lack such activity (Dostal et al., 2003). Despite this observed proteolytic activity, a comprehensive understanding of the functional characteristics of SAPs in these *Candida* species remains largely elusive. For instance, while *C. parapsilosis* harbors three *SAPP* genes (*SAPP1*, *SAPP2*, and *SAPP3*), and only *SAPP1* and *SAPP2* have been linked to extracellular protease activity (Singh et al., 2019). Interestingly, the deletion of *SAPP1* and *SAPP2* does not decrease pathogenicity, indicating that not all proteinases necessarily contribute to virulence (Singh et al., 2019). In this study, we identified 14 proteins within *C. auris* that contain the aspartic peptidase domain in *C. auris*. The functional analysis of the selected SAP genes (Sapa1 to Sapa7) revealed that only Sapa3 contributed to SAP activity in the YCB-BSA assay. The remaining SAPA genes appeared to have no evident *in vitro* functions, suggesting they might not play a role in *C. auris* pathogenicity. However, since we solely used BSA as a substrate, evaluating SAP activity with alternative substrates could be informative. Future research is also needed to delve into their potential pathobiological roles.

In a previous study, we made the intriguing observation that deleting key components of the cAMP/PKA pathway, Cyr1 or Tpk1/2, does not reduce the virulence of *C. auris*, despite growth defects at 37°C and increased stress sensitivity in the *cyr1*Δ and *tpk1*Δ *tpk2*Δ mutants (Kim et al., 2021). This led us to hypothesize that downstream factors positively regulated by the cAMP/PKA pathway might have a negative impact on the virulence of *C. auris*. For example, Tpk1/2 has been found to suppress the transition from the haploid to the diploid state, a ploidy switch known to enhance *C. auris* virulence (Kim et al., 2021). This study further establishes the critical role of the Tpk1/2-regulated expression and activity of Sapa3 in *C. auris* virulence. Therefore, although deleting Tpk1/Tpk2 itself does not affect pathogenicity, controlling downstream factors regulated by Tpk1/Tpk2 can reduce *C. auris* virulence, leading to this paradoxical scenario. To provide additional evidence, further experiments are needed to characterize the roles of Tpk1/Tpk2 downstream effectors in the pathogenicity of *C. auris*.

In conclusion, our study provides compelling evidence for the involvement of Sapa3 in SAP activity and the pathogenicity of *C. auris*. In *C. albicans*, SAPs play a pivotal role in breaking down a diverse range of cellular substrates. These substances encompass proteins associated with immunological responses and structural integrity, including but not limited to IgG heavy chains, α2-macroglobulin, C3 protein, β-lactoglobulin, lactoperoxidase, collagen, and fibronectin (Kaminishi et al., 1995). The proteolytic capability mediated by Sapa3 in *C. auris* hints at specialized mechanisms employed by this yeast to compromise the host’s immunological defenses and structural cohesion. Overall, our study highlights the role of Sapa3 as a major aspartyl proteinase involved in *C. auris* virulence.

## Data availability statement

The original contributions presented in the study are included in the article/**Supplementary Material**. Further inquiries can be directed to the corresponding author.

## Ethics statement

The animal study was approved by Institutional Animal Care and Use Committee of the Experimental Animal Center at Jeonbuk National University. The study was conducted in accordance with the local legislation and institutional requirements.

## Author contributions

J-SK: Data curation, Formal Analysis, Investigation, Methodology, Software, Validation, Visualization, Writing – original draft. K-TL: Data curation, Formal Analysis, Investigation, Methodology, Software, Resources, Writing – review & editing. Y-SB: Data curation, Formal Analysis, Resources, Writing – review & editing, Conceptualization, Funding acquisition, Project administration, Supervision.

## Supplementary material

Supplementary Figure 1 Construction and validation of gene deletion mutants complemented strain in *Candida auris*. **(A–G)** The schematic representations illustrate the homologous recombination strategies between the wild-type gene and the deletion cassette (left panels). Confirmation of transformants was achieved through diagnostic PCR (center panels) and further substantiated by Southern blot analysis (right panels). **(H)** Validation of the constructed complemented strains.

Supplementary Figure 2 Induction of filamentous growth using hydroxyurea (HU). Cells initially cultured overnight at 30°C were subcultured to OD_600 _of 0.8 in fresh YPD medium. Post-sub-culturing, they were resuspended in YPD broth containing 100 mM HU and incubated at 30°C for 24 hours. Following incubation, the cells were fixed with 10% formalin and stained using calcofluor white. Scale bars indicate 10 μm.

Supplementary Figure 3 Stress response and adaptation of *C. auris* SAP gene mutants. The figure presents a spot analysis of *C. auris* wild-type and SAP gene mutant strains under a range of stress conditions, including antifungal drugs, oxidative stress, osmotic challenges, cell wall and membrane perturbations, genotoxicity, heavy metals, ER stress, and acidic pH.

Supplementary Table 1
*C. auris* strains and primers used in this study.

Supplementary Table 2 SAPs blast analysis result.
