# Supplementary material for: Secreted aspartyl protease 3 regulated by the Ras/cAMP/PKA pathway promotes the virulence of Candida auris
Source: Front Cell Infect Microbiol. 2023 Sep 15;13:1257897. doi: 10.3389/fcimb.2023.1257897 (PMC10540861; doi:10.3389/fcimb.2023.1257897)
Supplement: Supplementary file 1 [file Table_1.pdf]

Table S1. ***C. auris*** **strains and primers used in this study**

**Strains used in this study**			
**Strain**	Genotype	**Parent**	**Reference**
**B8441**	Wild-type		(Munoz et al., 2018)
**YSBA21**	*cyr1*Δ::*NAT*	B8441	(Kim et al., 2021)
**YSBA24**	*tpk2*Δ::*NAT tpk1*Δ::*HYG*	YSBA17	(Kim et al., 2021)
**YSBA39**	*gpa2*Δ::*NAT*	B8441	(Kim et al., 2023)
**YSBA41**	*ras1*Δ::*NAT*	B8441	(Kim et al., 2023)
**YSBA46**	*gpr1*Δ::*NAT*	B8441	(Kim et al., 2023)
**YSBA61**	*cdc25*Δ::*NAT*	B8441	(Kim et al., 2023)
**YSBA68**	*gpa2*Δ::*NAT ras1*Δ::*HYG*	YSBA39	(Kim et al., 2023)
**YSBA77**	*sapa1*Δ::*NAT*	B8441	This study
**YSBA81**	*sapa2*Δ::*NAT*	B8441	This study
**YSBA91**	*sapa6*Δ::*NAT*	B8441	This study
**YSBA119**	*sapa3*Δ::*NAT*	B8441	This study
**YSBA123**	*sapa3*Δ::*SAPA3-HYG*	YSBA119	This study
**YSBA125**	*sapa5*Δ::*NAT*	B8441	This study
**YSBA127**	*sapa7*Δ::*NAT*	B8441	This study
**YSBA135**	*sapa4*Δ::*NAT*	B8441	This study

Primers used in this study

Name	Primer description	Sequence (5' to 3')
**B15692**	*SAPA1* 5'-flanking region primer L1	GGCTGTAGATGAGCCGTAGG
**B15693**	*SAPA1* 5'-flanking region primer L2	TTCAGATCCACTAGTTCTAGCGAGTGTTGTTGGTTGATGA
**B15694**	*SAPA1* 3'-flanking region primer R1	TTTCCAGAATTTCACTCTTAATTTTTTTTAGATTTTTA
**B15695**	*SAPA1* 3'-flanking region primer R2	GGGTATGTGCTTGCTTGGTT
**B15696**	*SAPA1* 5'-screening primer SO	AGGTCACCCACAGGAGTCAC
**B15697**	*SAPA1* 3'-screening primer SO2	TGTGTTGGTGCCTTTGACAT
**B15698**	*SAPA1* Southern blot probe primer PO	AAGAGGCGCCTTCCTCAGGC
**B15721**	*SAPA1* Internal screening primer LP	TGTATGGTCCCTCCGAGAAG
**B15722**	*SAPA1* Internal screening primer RP	AAGAGACACTGGCCCTGAGA
**B16032**	*SAPA2* 5'-flanking region primer L1	AACTTGCCAGCCAAGCTTTA
**B16033**	*SAPA2* 5'-flanking region primer L2	TTCAGATCCACTAGTTCTAGGAAGATTGATTCAAAAGACT
**B16034**	*SAPA2* 3'-flanking region primer R1	TTTCCAGAATTTCACTCTTAAAAAGTTTCTATGCCCAATA
**B16035**	*SAPA2* 3'-flanking region primer R2	GGAAGGTCGGTGATTGAAAA
**B16036**	*SAPA2* 5'-screening primer SO	CCTCAACATTTTGCATGACG
**B16037**	*SAPA2* 3'-screening primer SO2	GGACGGAACTCGTTGTTGAT
**B16038**	*SAPA2* Southern blot probe primer PO	TTTGTCGAGAGCCAGAGAGT
**B16039**	*SAPA2* Internal screening primer LP	TCTCTTTTGCCTTGGCTTGT
**B16040**	*SAPA2* Internal screening primer RP	AATAGGACGCCTTTGGTGTG
**B16580**	*SAPA3* 5'-flanking region primer L1	AAACCCTGCCCTCCAGTAAT
**B16581**	*SAPA3* 5'-flanking region primer L2	TTCAGATCCACTAGTTCTAGGGCGAGGGTGAAGATGGTCA
**B16582**	*SAPA3* 3'-flanking region primer R1	TTTCCAGAATTTCACTCTTAATGCTTCAGGTAAGCACCAC
**B16583**	*SAPA3* 3'-flanking region primer R2	TGATCAACCAGGACGTGTGT
**B16584**	*SAPA3* 5'-screening primer SO	AACCGAGAGATGCAAGGAGA
**B16585**	*SAPA3* 3'-screening primer SO2	TTCCTTGAGAGACCCTGTGG
**B16588**	*SAPA3* Southern blot probe primer PO	GTGACCTTGTCGTGCTGAGA
**B16586**	*SAPA3* Internal screening primer LP	TCTCAGCACGACAAGGTCAC
**B16587**	*SAPA3* Internal screening primer RP	GGCACCCAAAGAACACACTT
**B16636**	*SAPA5* 5'-flanking region primer L1	CTTATATGCCTTGCGGTGGT
**B16637**	*SAPA5* 5'-flanking region primer L2	TTCAGATCCACTAGTTCTAGTGTTTGATAGCGATGAAAAT
**B16638**	*SAPA5* 3'-flanking region primer R1	TTTCCAGAATTTCACTCTTAGCGCACGTTCGATCGATTGC
**B16639**	*SAPA5* 3'-flanking region primer R2	GATCCGGGTTGCACACTAC
**B16640**	*SAPA5* 5'-screening primer SO	GTGAGGAAAGGGAATGGACA
**B16641**	*SAPA5* 3'-screening primer SO2	TGCCCACAGGCTGTATGTAA
**B16644**	*SAPA5* Southern blot probe primer PO	ACTTGGAGTTGCCCACATTC
**B16642**	*SAPA5* Internal screening primer LP	GCTTTGACGGAGAGACCTTG
**B16643**	*SAPA5* Internal screening primer RP	CAAAATGTAGCGGTCCTGGT
**B16625**	*SAPA5* 5'-flanking region primer L1	GAGAAGCGCATTTACCGAAG
**B16626**	*SAPA4* 5'-flanking region primer L2	TTCAGATCCACTAGTTCTAGTGAGGATGATTTTTGGAAAG
**B16627**	*SAPA4* 3'-flanking region primer R1	TTTCCAGAATTTCACTCTTACAGTGGATGTTGGAGTCTAA

**B16628**	*SAPA4* 3'-flanking region primer R2	TTACTAAGATGCCCGCCTTG
**B16629**	*SAPA4* 5'-screening primer SO	ATCAATTTCCGCAATGCTTC
**B16630**	*SAPA4* 3'-screening primer SO2	CGCTTGAACATTTCGACTCA
**B16633**	*SAPA4* Southern blot probe primer PO	GGTCAGAAGAGCCAGTGTCC
**B16631**	*SAPA4* Internal screening primer LP	AGAACCGTTGCTCCTCTCAA
**B16632**	*SAPA4* Internal screening primer RP	TCGTAGTGTGGATGCAGCTC
**B18843**	*SAPA6* 5'-flanking region primer L1	CAGAAGCCAATGATCAGCAA
**B18844**	*SAPA6* 5'-flanking region primer L2	TTCAGATCCACTAGTTCTAGTGTATTTAGATCTTATTCCC
**B18845**	*SAPA6* 3'-flanking region primer R1	TTTCCAGAATTTCACTCTTAAGGATTGATAGATTTTCACA
**B18846**	*SAPA6* 3'-flanking region primer R2	GATTGGCTCACCGAGAAGAA
**B16368**	*SAPA6* 5'-screening primer SO	TCTGAAATCCCCAAGATTCG
**B13689**	*SAPA6* 3'-screening primer SO2	TTTGGTGCTCGACAGACAAG
**B18847**	*SAPA6* Southern blot probe primer PO	CCATCTCGCTTGACAAAGGT
**B16370**	*SAPA6* Internal screening primer LP	AACGAATCCATCTCGTCCAC
**B16371**	*SAPA6* Internal screening primer RP	GCACACTCACCAACACCATC
**B16943**	*SAPA7* 5'-flanking region primer L1	TTGTGGCCATGATGAGAAAA
**B16944**	*SAPA7* 5'-flanking region primer L2	TTCAGATCCACTAGTTCTAGGTTGGAAAAGAGAAGACGGA
**B16945**	*SAPA7* 3'-flanking region primer R1	TTTCCAGAATTTCACTCTTATACAATAAATATATATGTAT
**B16946**	*SAPA7* 3'-flanking region primer R2	CGTCCAAGGCCTATGACAAT
**B16947**	*SAPA7* 5'-screening primer SO	GCCCTTACAACGTACCCTCA
**B16948**	*SAPA7* 3'-screening primer SO2	GCCGATACCCTTGTCTACCA
**B16951**	*SAPA7* Southern blot probe primer PO	TGTCGCCGTACAAGACGTAG
**B16949**	*SAPA7* Internal screening primer LP	GAGAGACGACGACACGTTGA
**B16950**	*SAPA7* Internal screening primer RP	CAAGAGCACGTTGGTTCTGA
**B11103**	pV1025 forward-extended primer	CTAGAACTAGTGGATCTGAA
**B11104**	pV1025 reverse-extended primer	TAAGAGTGAAATTCTGGAAA
**B11105**	*NAT* split primer 1	CCATTGACTAAGGTTTTCCC
**B11106**	*NAT* split primer 2	TTCAGTAGCCAAACCCATC
**B11107**	pV1025 Diagnostic screening primer 1	TCAGTGGCAAATCCTAACC
**B11108**	pV1025 Diagnostic screening primer 2	AGAGAAAATACCCGTGACG
**B17342**	CLP for *SAPA3* complementation	TTGTTTACTGAGGACAAGGA
**B17343**	CRP for *SAPA3* complementation	TATTTCCTTGAGAGACCCTG
**B17347**	Screening primer for *SAPA3* complementation	GCCTACATTTGCCAGGAAAG
**B17344**	*SAPA3* sequencing primer 1	TGTATCTATAATAAGATTCC
**B17345**	*SAPA3* sequencing primer 2	TTTTTCCATCCAGTACTTGG
**B11749**	*ACT1* qRT-PCR primer 1	TTGCTCCTGAAGAACACCCT
**B11750**	*ACT1* qRT-PCR primer 2	GCAGGAACGTTGAAGGTCTC
**B17310**	*SAPA3* qRT-PCR primer 1	AGGCGAGCCTACTTACGACA
**B17311**	*SAPA3* qRT-PCR primer 2	CTCAGAGCCCAAGAACAAGG

References

Kim, J.S., Lee, K.T., and Bahn, Y.S. (2023). Deciphering the regulatory mechanisms of the cAMP/PKA pathway and their roles in the pathogenicity of *Candida auris*. *Microbiol Spectr*. doi: 10.1128/spectrum.02152-23.

Kim, J.S., Lee, K.T., Lee, M.H., Cheong, E., and Bahn, Y.S. (2021). Adenylyl cyclase and protein kinase A play redundant and distinct roles in growth, differentiation, antifungal drug resistance, and pathogenicity of *Candida auris*. *mBio* 12**,** e0272921. doi: 10.1128/mBio.02729-21

Munoz, J.F., Gade, L., Chow, N.A., Loparev, V.N., Juieng, P., Berkow, E.L., Farrer, R.A., Litvintseva, A.P., and Cuomo, C.A. (2018). Genomic insights into multidrug-resistance, mating and virulence in *Candida auris* and related emerging species. *Nat Commun* 9**,** 5346. doi: 10.1038/s41467-018-07779-6
