# Supplementary figures and images for: Secreted aspartyl protease 3 regulated by the Ras/cAMP/PKA pathway promotes the virulence of Candida auris
Source: Front Cell Infect Microbiol. 2023 Sep 15;13:1257897. doi: 10.3389/fcimb.2023.1257897 (PMC10540861; doi:10.3389/fcimb.2023.1257897)

Figure S1 (Kim et al.)

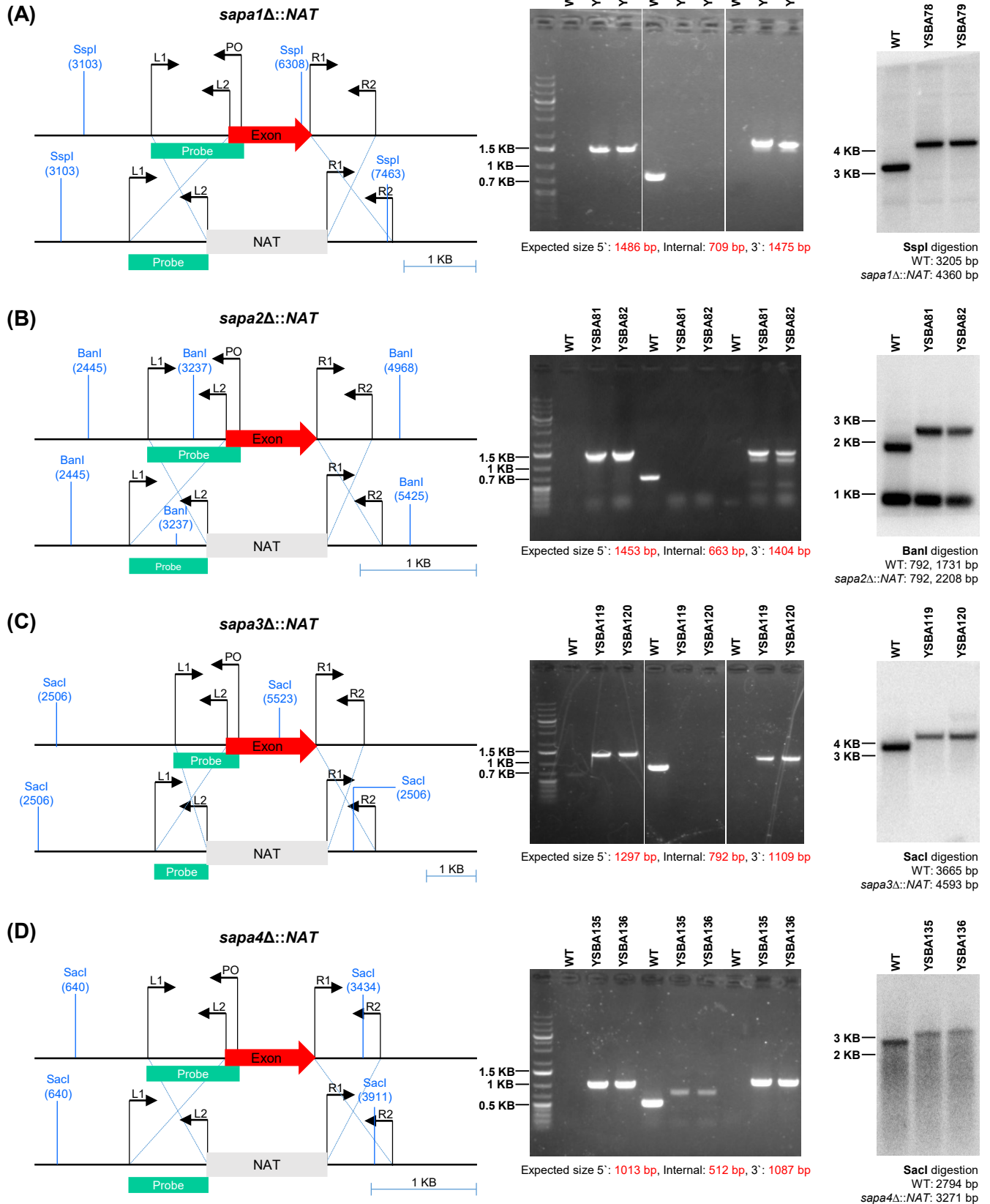

Figure S1 (Kim et al.)

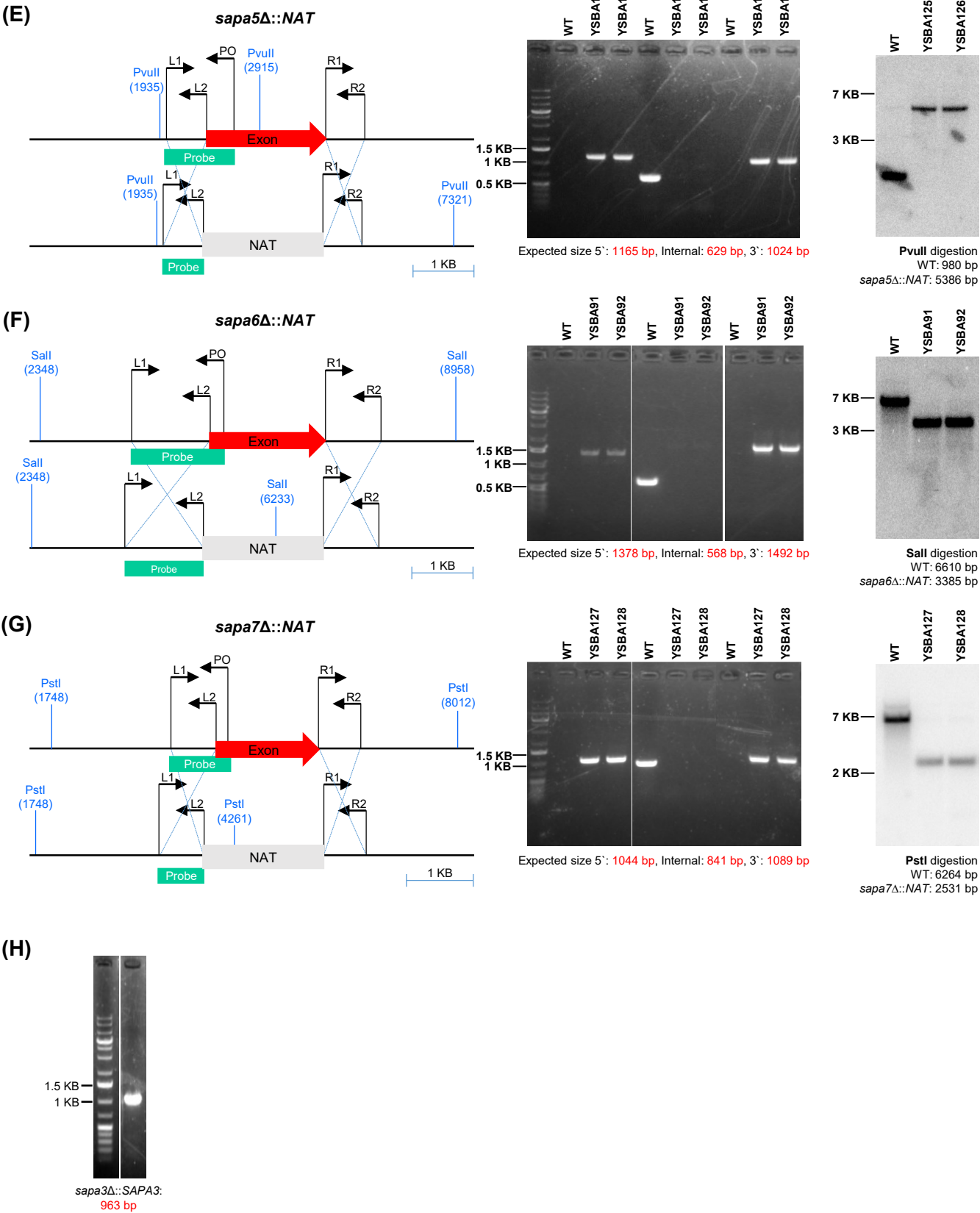

**Figure S2 (Kim et al.)**

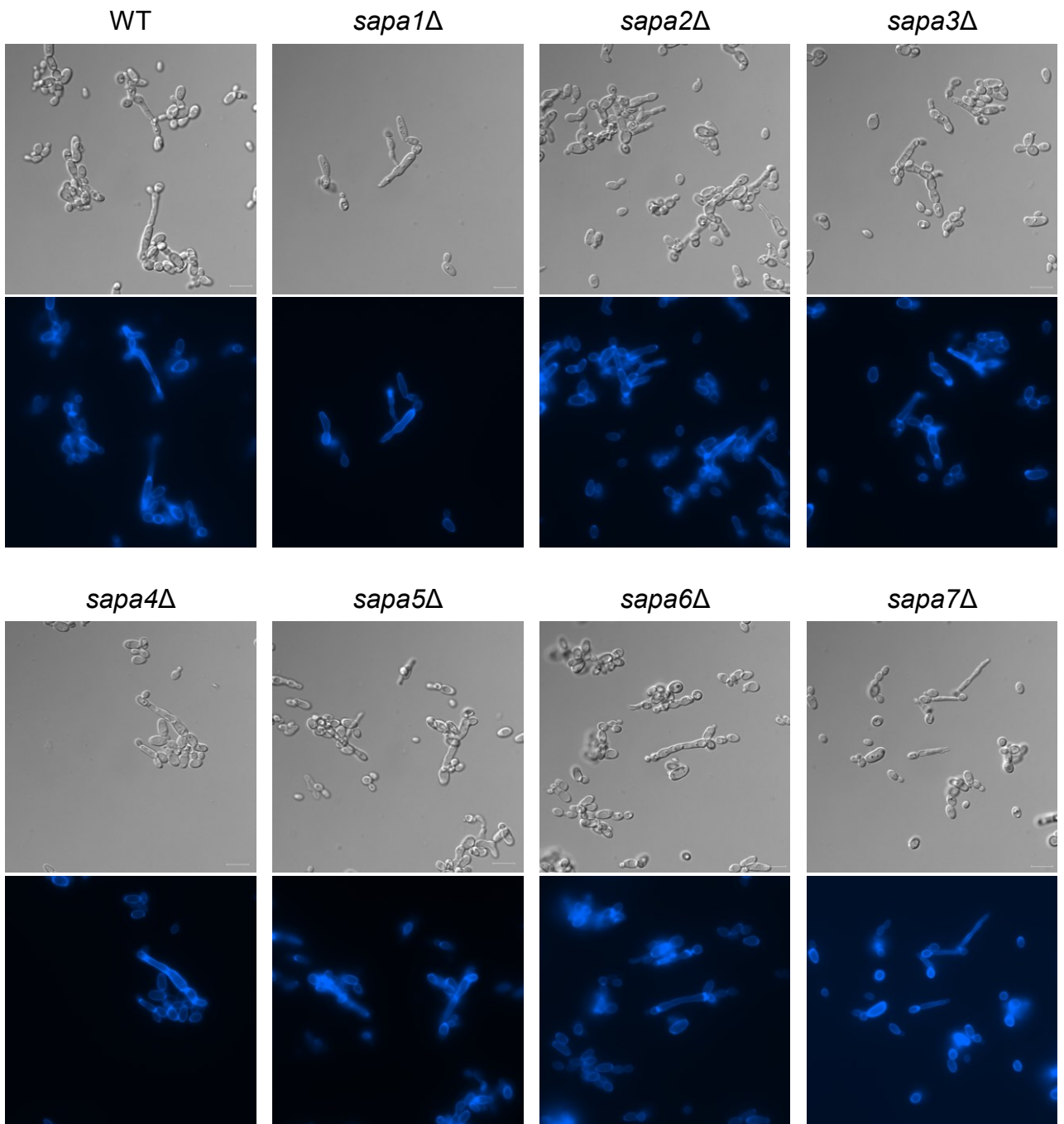

Figure S3 (Kim et al.)

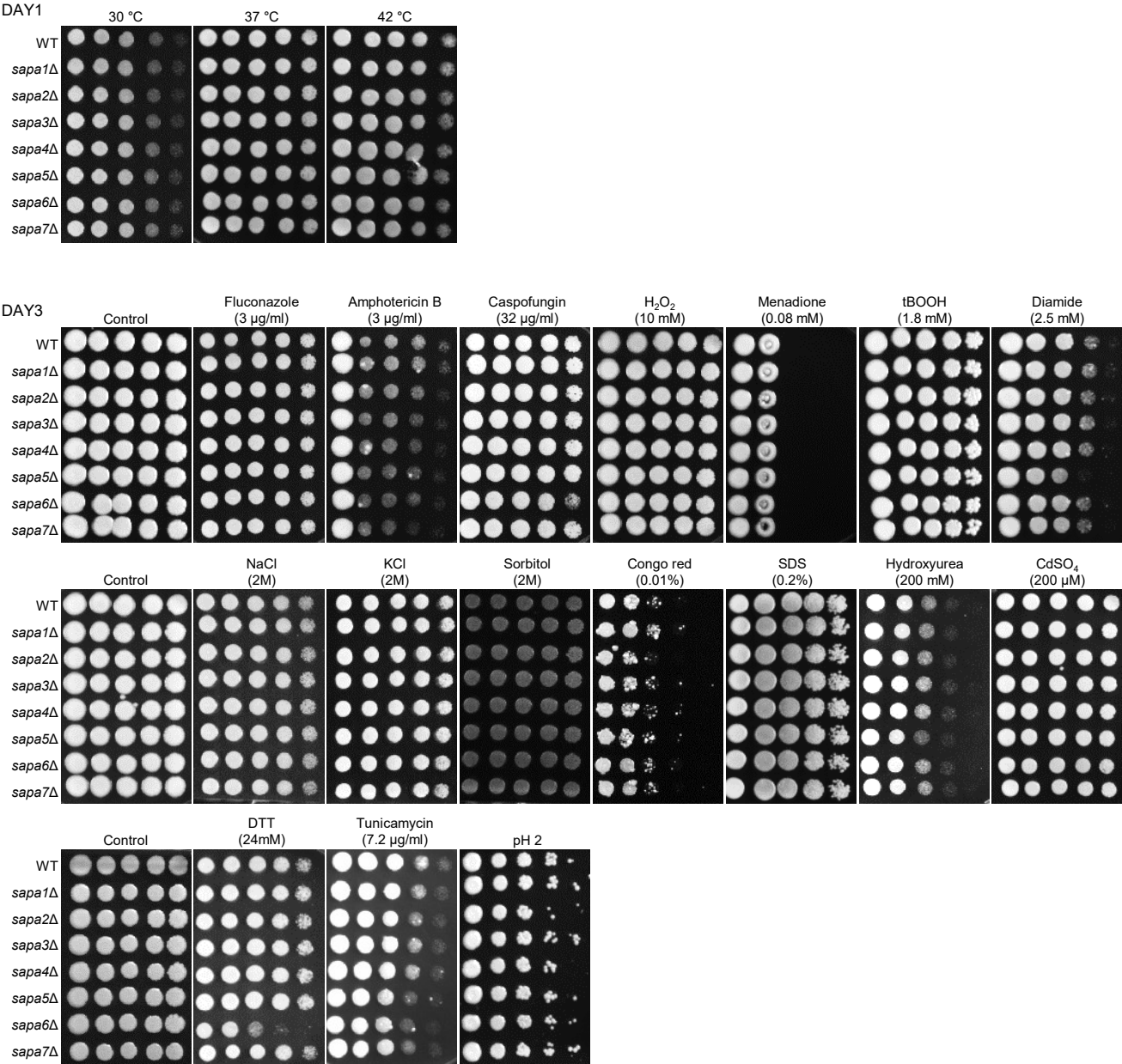

Supplement: Supplementary Figure 1 — Construction and validation of gene deletion mutants complemented strain in Candida auris. (A–G) The schematic representations illustrate the homologous recombination strategies between the wild-type gene and the deletion cassette (left panels). Confirmation of transformants was achieved through diagnostic PCR (center panels) and further substantiated by Southern blot analysis (right panels). (H) Validation of the constructed complemented strains. [file Image_1.pdf]
